# Acute toxicity of intratracheal arsenic trioxide instillation in rat lungs

**DOI:** 10.1002/jat.3841

**Published:** 2019-07-18

**Authors:** Su Mingxing, Wang Haiying, Sun Congsong, Yuan Chunyu, Chao Liu, Qiang Wang

**Affiliations:** ^1^ Chinese People's Liberation Army Center of Disease Control and Prevention Beijing China

**Keywords:** acute toxicity, arsenic trioxide, dynamic change, intratracheal instillation, lung

## Abstract

This study investigated the acute toxicity of different concentrations of arsenic trioxide (As_2_O_3_; ATO) on rat lungs. In total, 160 Wistar rats were randomly divided into the control, low‐, medium‐ and high‐dose groups, which were exposed to 0, 0.16, 1.60 and 16 μg/kg of ATO by intratracheal instillation, respectively. Samples were collected at 6, 12, 24, 48 and 72 hours after exposure and the dynamic changes indicative of acute lung toxicity were monitored. Compared with the control group, the exposure groups exhibited significant changes such as increased lung water content ratio and protein concentration in the bronchoalveolar lavage fluid, pulmonary interstitial thickening, cell membrane edema, increased inflammatory factor concentration, JNK and P38 were significantly activated, and the degree of phosphorylation was increased. Furthermore, all the changes in the exposure groups were exposure concentration‐dependent. ATO respiratory tract exposure can cause restrictive ventilatory disturbance in rats, and the degree of injury is exposure concentration‐dependent.

## Abstract 2

To investigate the acute toxicity of arsenic trioxide on lungs, 160 Wistar rats were randomly divided into the control group, low‐, medium‐ and high‐dose groups. The results showed that there were significant changes in lung water content ratio, bronchoalveolar lavage fluid protein concentration, pulmonary interstitial thickening, cell membrane edema, inflammatory factor concentration, JNK and p38 phosphorylation in the exposed group. Collectively, acute atmospheric arsenic exposure may be associated with a risk of inflammatory lung injury, which is a health concern that deserves more attention.

## 1 INTRODUCTION

Air pollution is a global problem that cannot be ignored. It is a considerable threat to human health, particularly that of the respiratory tract (Tie et al., 2017; Urbancok, Payne, & Webster, 2017). The main air pollutant is particulate matter, and the analysis of its composition revealed the presence of arsenic (As) as one of the most potentially concerning pollutant components (Bennett & Benson, 2005; Tan et al., 2017). At the same time, compared with the European Commission Air Quality Standards (European Commission, 2005), As was found to be one of the most serious pollutants exceeding the standard recommended levels. At present, the research into As exposure by inhalation exposure is related to occupational toxicology. Studies in occupational populations have found that As exposure can lead to pulmonary edema, pulmonary interstitial fibrosis, alveolar wall edema, pulmonary capillary occlusion, and pathological changes in lamellar body reduction. However, the exposure to As caused by air pollution, particularly the acute exposure to As under extremely heavy haze, is still unclear on the human lung. Every day, the human body exchanges 11 000 L of air through respiration and the lungs are continuously exposed to As contaminants. Epidemiological data show that a variety of acute and chronic diseases is related to air pollution (Gu et al., 2017). Particularly in heavily polluted conditions, the number of outpatients with respiratory diseases has increased dramatically. However, the relationship between the increased respiratory diseases and air As needs to be studied further.

As belongs to the group 1 carcinogens established by the International Agency for Research on Cancer (IARC) and has a definite carcinogenicity to the skin and lungs (IARC, 1987; Khanjani, Jafarnejad, & Tavakkoli, 2017). Presently, the As‐induced hazard to the human body has been fully studied (Aballay, Díaz, Francisca, & Muñoz, 2012; Baastrup et al., 2008). However, these studies have mainly focused on gastrointestinal tract exposure, and the occupational population is the main research area. It was also found that As in atmospheric particulate matter mainly exists in the form of arsenic trioxide (As^3+^, As_2_O_3_ [ATO]). At the same time, it is worth noting that the biological toxicity of As^3+^ is much higher than that of As^5+^. Therefore, the establishment of animal models and a thorough study of the effects of respiratory ATO exposure are of great significance.

## 2 MATERIALS AND METHODS

### 2.1 Reagents and equipment

ATO (100 mg/mL; no. 14041) was purchased from the China Institute of Metrology. Chloral hydrate (Sino Pharm; batch no. 20150303), enzyme‐linked immunosorbent assay kits for tumor necrosis factor (TNF)‐α (batch no. 2016/09), interleukin (IL)‐6 (batch no. 2016/09) and IL‐10 (batch no. 2016/09; all Unionhonest) were purchased from the Beijing Laboratory Biology Technology Co., Ltd. Coomassie brilliant blue G‐250 (JKHD; batch no. 31650309), and the spectrophotometer (U3010; Hitachi) were provided by the Chinese People's Liberation Army Center of Disease Control and Prevention.

### 2.2 Animal groups

In total, 160 specific pathogen‐free Wistar rats (qualification no. 0023373), weighing 100‐150 g, were purchased from the Experimental Animal Center of the Military Medical Science Academy of the Chinese People's Liberation Army. The rats were randomly divided into control (CON), low‐, medium‐ and high‐dose (LD, MD and HD, respectively) groups using a random number table. In addition, each group was further randomly divided into five subgroups of eight rats each, which were sampled 6, 12, 24, 48 and 72 hours after exposure. The rats were maintained in a barrier environment for 1 week of quarantine and acclimatization at a temperature of 22 ± 2°C, relative humidity of 55% ± 5%, on a 12‐hour light/dark cycle, and food and water access was ad libitum.

### 2.3 Animal model establishment

Through literature research on the As content in the air of different countries and regions, with reference to the As exposure concentration of the occupational population, combined with the tidal volume of the rat, we set LD, MD and HD groups to give 0.16, 1.60 and 16 mg/mL ATO, respectively, to simulate As inhalation exposure in mild, severe and extreme heavy haze conditions. ATO was diluted to different concentrations with ultrapure water, and the pH value was adjusted to 7.4 before use. Rats were anesthetized with a 0.75 mL/kg chloral hydrate intraperitoneal injection. The trachea of each rat was instilled with 0.06 mL of ATO at 0.16, 1.6 and 16 μg/kg daily for 3 days or saline (CON) alone. Following the last exposure, samples were collected at 6, 12, 24, 48 and 72 hours. Animal experiments have been approved by ethics committees and they comply with the relevant regulations.

### 2.4 Inflammatory factor measurement

The blood samples were collected from the abdominal aorta using a vacuum blood vessel tube, centrifuged at 2504 g for 10 minutes at 4°C and then the serum was collected and analyzed in time to avoid the repetitive freezing and thawing caused by sample storage. TNF‐α, IL‐6 and IL‐10 levels were detected according to the instructions for the enzyme‐linked immunosorbent assay kits.

### 2.5 Measurement of total protein concentration in bronchoalveolar lavage fluid

The rats were exsanguinated through the abdominal aorta, the blood drained, the chest was opened and then the right lung was clamped using hemostatic forceps. After tracheal intubation, the left lung was repeatedly lavaged three times with 3 mL 4°C normal saline for 5 seconds. The bronchoalveolar lavage fluid (BALF) was recovered and placed in a 15 mL centrifuge tube, centrifuged at 352 g for 10 minutes at 4°C, the supernatant was collected, and then the total protein concentration was determined using the Coomassie brilliant blue G250 method.

### 2.6 Water content ratio measurement

The left first lung was harvested, weighed immediately, placed in an oven at 80°C until the weight was constant and then the final constant weight was subtracted from the initial weight to calculate the water content ratio.

### 2.7 Pathological specimens

The second left lung was harvested, fixed by perfusion with 4% formalin, dehydrated, cleared to transparency, embedded in paraffin, sectioned (4 μm) and then hematoxylin and eosin (H&E) stained. The sections were observed under a light microscope, focusing on pulmonary edema, protein exudation, neutrophils, bleeding and bronchial epithelial change, and then images were captured.

### 2.8 Western blot analysis

Lung tissues were homogenized in RIPA buffer plus protease inhibitors cocktail (Roche). Equivalent amounts of total protein containing 10 μg proteins were loaded on a single track of 12% sodium dodecyl sulfate‐polyacrylamide gel electrophoresis and transferred by electroblotting on to nitrocellulose membrane. The blots were blocked by using 5% blocking reagent in a solution of Tris‐buffered salt with Tween‐20 for 1 hour at room temperature, and then incubated with primary antibody (1:1000) overnight at 4°C. After being washed with Tris‐buffered salt with Tween‐20, the blots were incubated in secondary antibody conjugated horseradish peroxide (1:1000) for 1 hour at 37°C. After extensive washing, the complexes were visualized using West Pico chemiluminescent kit (Pierce).

### 2.9 Statistical analyses

The data were entered using the Microsoft EXCEL 2007 program and analyzed using SPSS 18.0 (IBM Corp.). The results are expressed as the mean ± standard deviation. Groups were compared using a one‐way analysis of variance and a *P* < .05 was considered statistically significant.

## 3 RESULTS

### 3.1 Dynamic changes in lung water content ratio in rats

**Figure 1:**
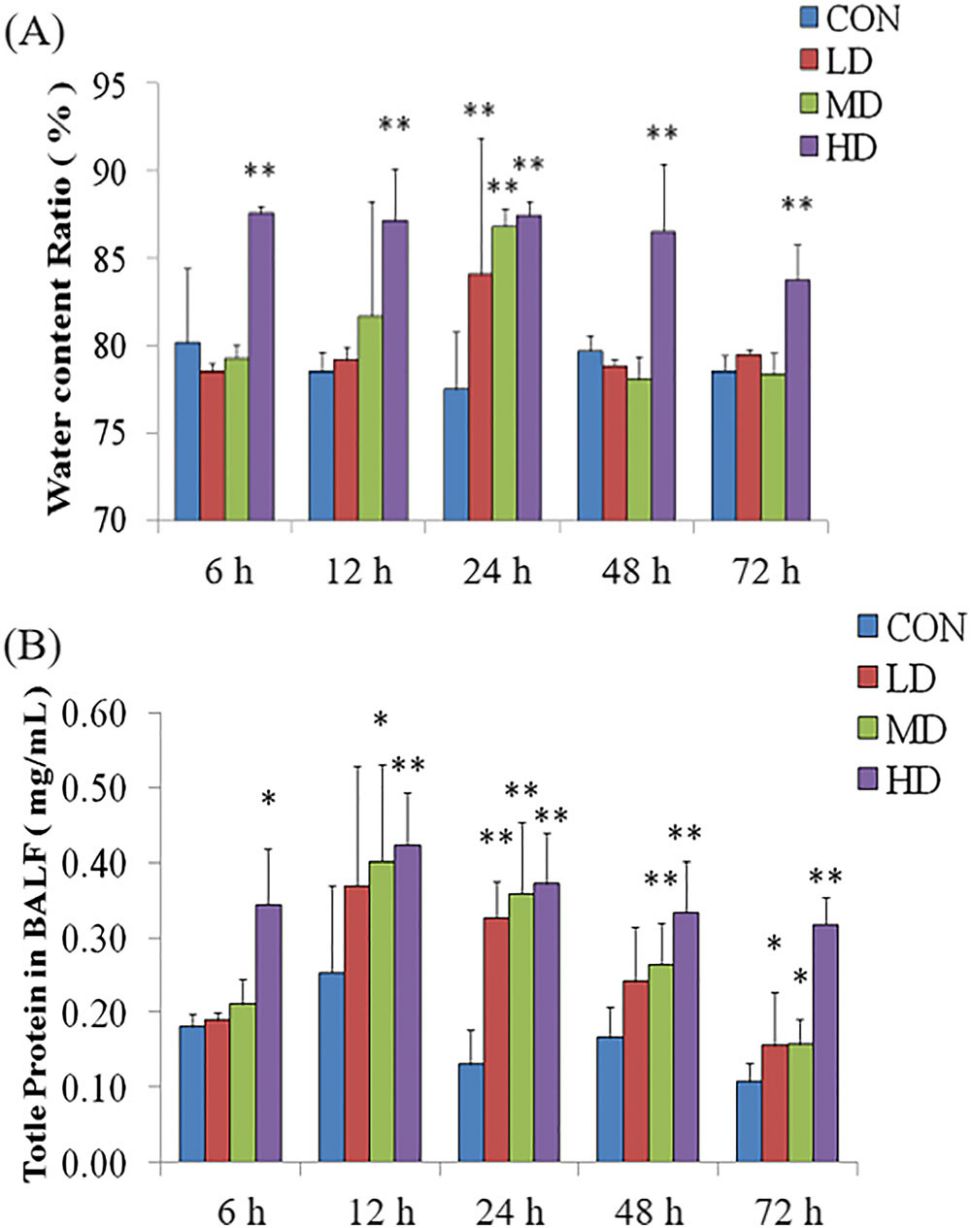
Dynamic changes in lung water content ratio and total protein concentration in BALF in rats. A, Dynamic changes in lung water content ratio of lung tissues after ATO exposure induced lung injury. Changes included the increase in water content ratio. B, Dynamic changes of total protein concentration in BALF after ATO exposure induced lung injury. Changes included the increase of total protein concentration in BALF. *P < .05 and **P < .01, compared with CON. ATO, arsenic trioxide; BALF, bronchoalveolar lavage fluid; CON, control group; HD, high‐dose group; LD, low‐dose group; MD, medium‐dose group [Colour figure can be viewed at https://wileyonlinelibrary.com]

Figure 1A shows that there was a significant increase in the lung water content ratio. Compared with the CON group, the LD and MD groups showed a significant increase in the lung water content only at 24 hours. Moreover, the HD group showed a significant increase in the water content ratio at 6 hours and this persisted for more than 72 hours (*P* < .01).

### 3.2 Dynamic changes in total protein concentration of bronchoalveolar lavage fluid in rats

Figure 1B shows that the total protein concentration in the BALF was increased. Compared with the CON group, the BALF total protein level of the LD group only appeared to increase significantly at 24 hours (*P* < .01). In the MD group, the level increased significantly from 12 hours (*P* < .05), exceeded 72 hours, and was significantly higher at 24 and 48 hours (*P* < .01). The HD group level increased significantly at 6 hours (*P* < .05) and then increased further for more than 72 hours (*P* < .01).

### 3.3 Dynamic changes in alveolar structural pathologies in rats

**Figure 2:**
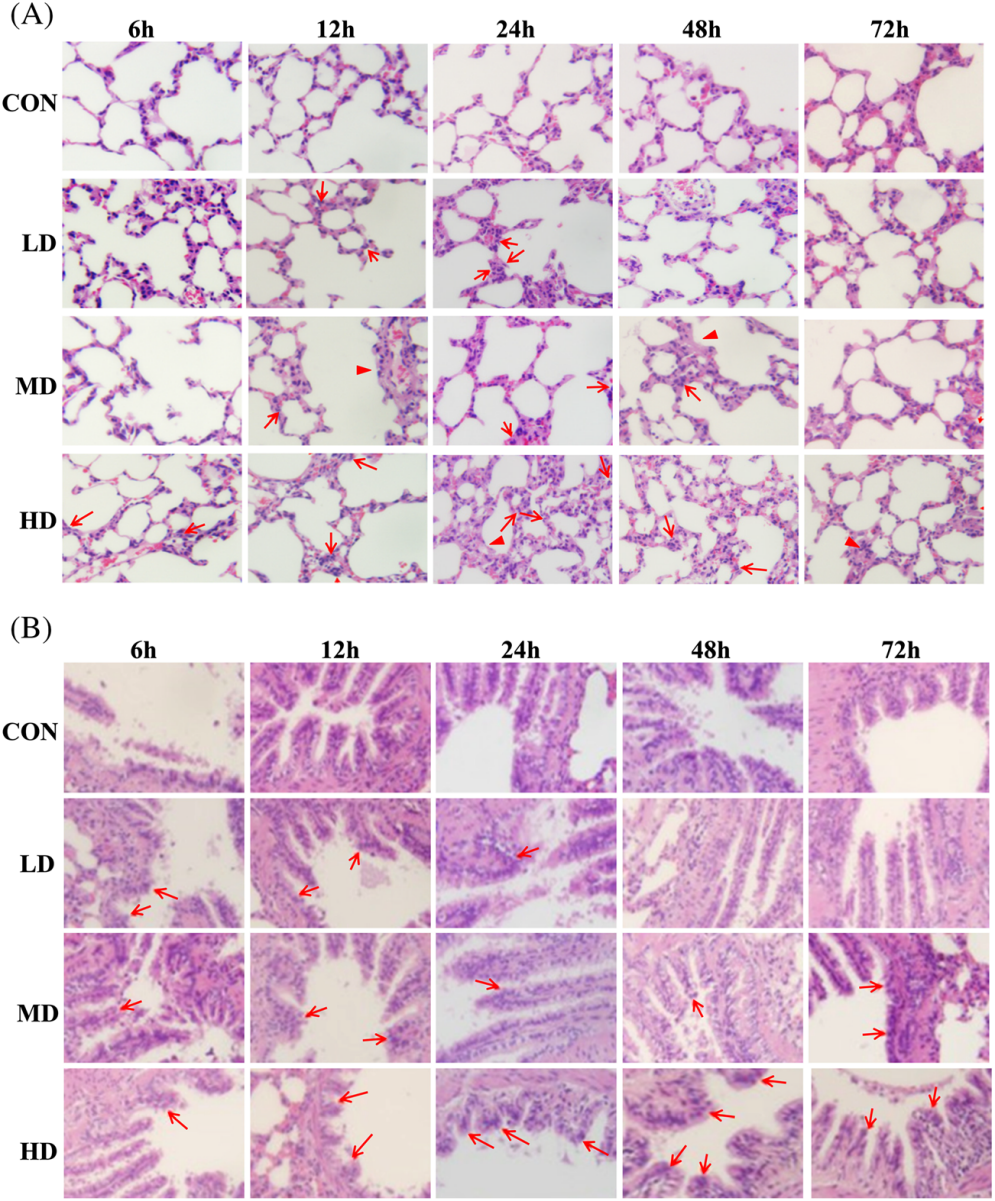
Dynamic changes of lung tissue pathologies in rats. A, Dynamic changes of alveolar structure pathologies of lung tissues after ATO exposure induced lung injury. These changes included pulmonary interstitial thickening, alveolar structural damage, cell membrane edema, inflammatory cell infiltration and focal lesions of fibrosis. ▲, pulmonary interstitial thickening; ↑, inflammatory cell infiltration. B, Dynamic changes of bronchioli terminales pathologies of lung tissues after ATO exposure induced lung injury. These changes included damage to bronchial cilia and upward movement of columnar nuclei. ↑, upward of nuclear. Hematoxylin and eosin staining for lung, 40×. ATO, arsenic trioxide; CON, control group; HD, high‐dose group; LD, low‐dose group; MD, medium‐dose group

The H&E staining illustrated in Figure 2A shows that compared with the CON group there was significant edema in the alveolar structure after ATO exposure. The LD group showed inflammatory cell infiltration, and destruction of the alveolar structure was only observed at 24 hours. The MD group also showed inflammatory cell infiltration after 6 hours, and the edema and damaged alveolar structure were evident at 24 hours and gradually reduced for up to 72 hours. In the HD group, inflammatory cell infiltration appeared after exposure for 6 hours. After 12 hours, the integrity of the alveoli began to exhibit damage, interstitial thickening, alveolar wall edema and thickening, and inflammatory cell infiltration, which persisted for up to 72 hours. The pathological changes were gradually eased off after that.

### 3.4 Dynamic changes in terminal bronchiolar pathologies in rats

The H&E staining illustrated in Figure 2B shows that compared with the CON group, the exposure groups exhibited no obvious mucus exudation or occasional infiltration of inflammatory cells, but significant damage to the terminal bronchiole epithelia was observed using the optical microscope. The LD group exhibited bronchial cilium adhesion, lodging and shedding, and the columnar nuclei moved upward while an obvious improvement was found after the 48‐hour exposure. In the MD and HD group, a 6‐hour exposure caused similar injury, but the damage was more serious after 12 hours, and inflammatory cell infiltration was discovered, which did not improve significantly over 72 hours.

### 3.5 Dynamic changes in inflammatory factor concentration in rats

**Figure 3:**
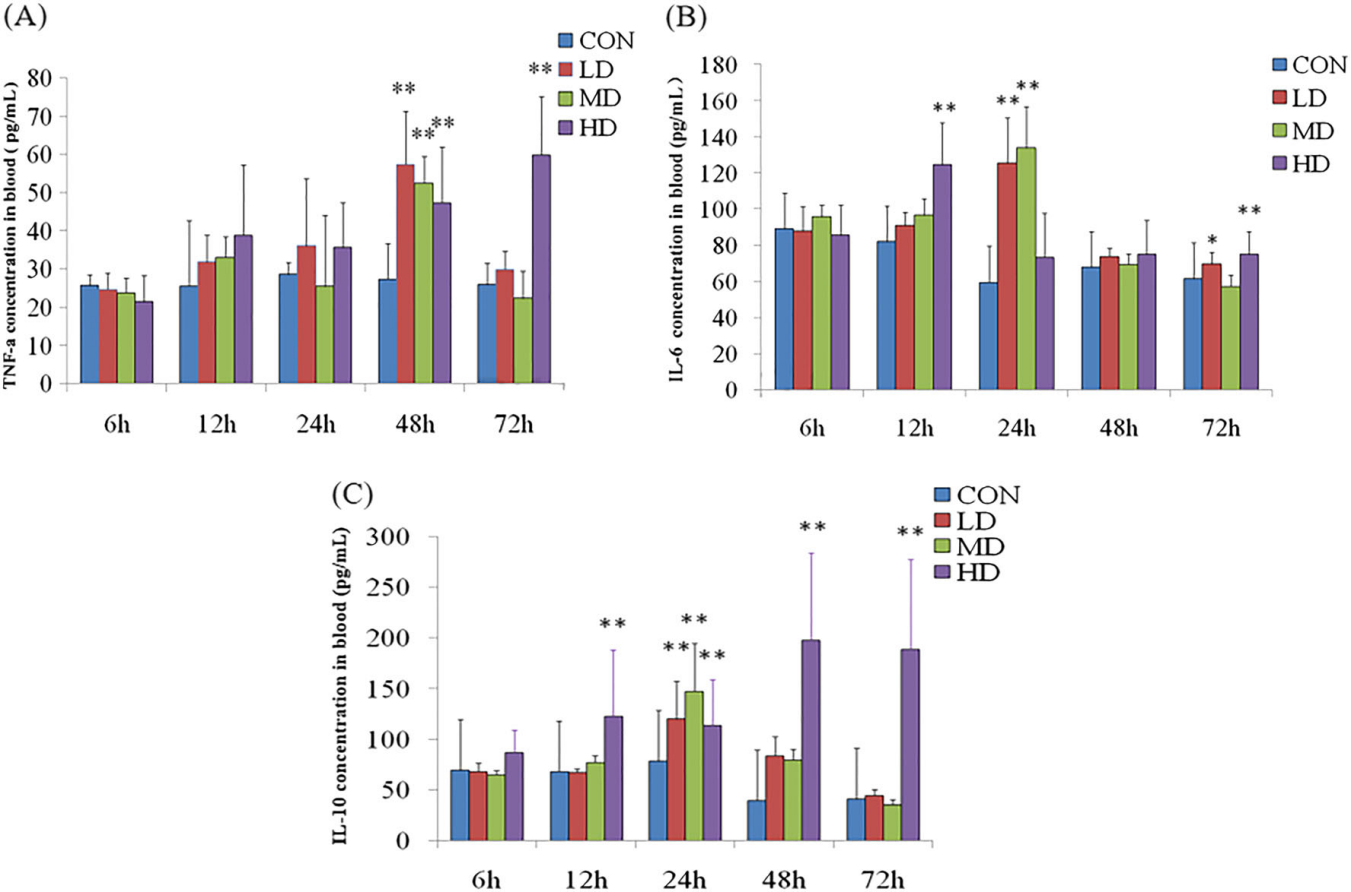
Dynamic changes of inflammatory factors concentration in rats. A, TNF‐α. B, IL‐6. C, IL‐10. Dynamic changes in serum TNF‐α, IL‐6 and IL‐10 after ATO exposure‐induced lung injury. *P < .05 and **P < .01 compared with CON. ATO, arsenic trioxide; CON, control group; HD, high‐dose group; IL, interleukin; LD, low‐dose group; MD, medium‐dose group; TNF, tumor necrosis factor [Colour figure can be viewed at https://wileyonlinelibrary.com]

Figure 3A shows that compared with the CON group, the LD and MD groups only showed a significant increase in serum TNF‐α at 48 hours (*P* < .01). In the HD group, the level also significantly increased (*P* < .01) at 48 hours, but did not recover after 72 hours. Figure 3B shows a significant increase in the serum concentration of IL‐6 in the LD and MD groups compared with that in the CON group only at 24 hours (*P* < .01). In the HD group, the IL‐6 concentration significantly increased (*P* < .01) at 12 hours and then recovered. However, the level appeared to increase significantly at 72 hours again (*P* < .01). Figure 3C shows a significant increase in the serum concentration of IL‐10 in the LD and MD groups compared with that in the CON group only at 24 hours (*P* < .01). Similarly, the HD group showed a significant increase at 12 hours (*P* < .01) but did not recover after 72 hours.

### 3.6 Effect of arsenic trioxide exposure on the mitogen‐activated protein kinase pathway

**Figure 4:**
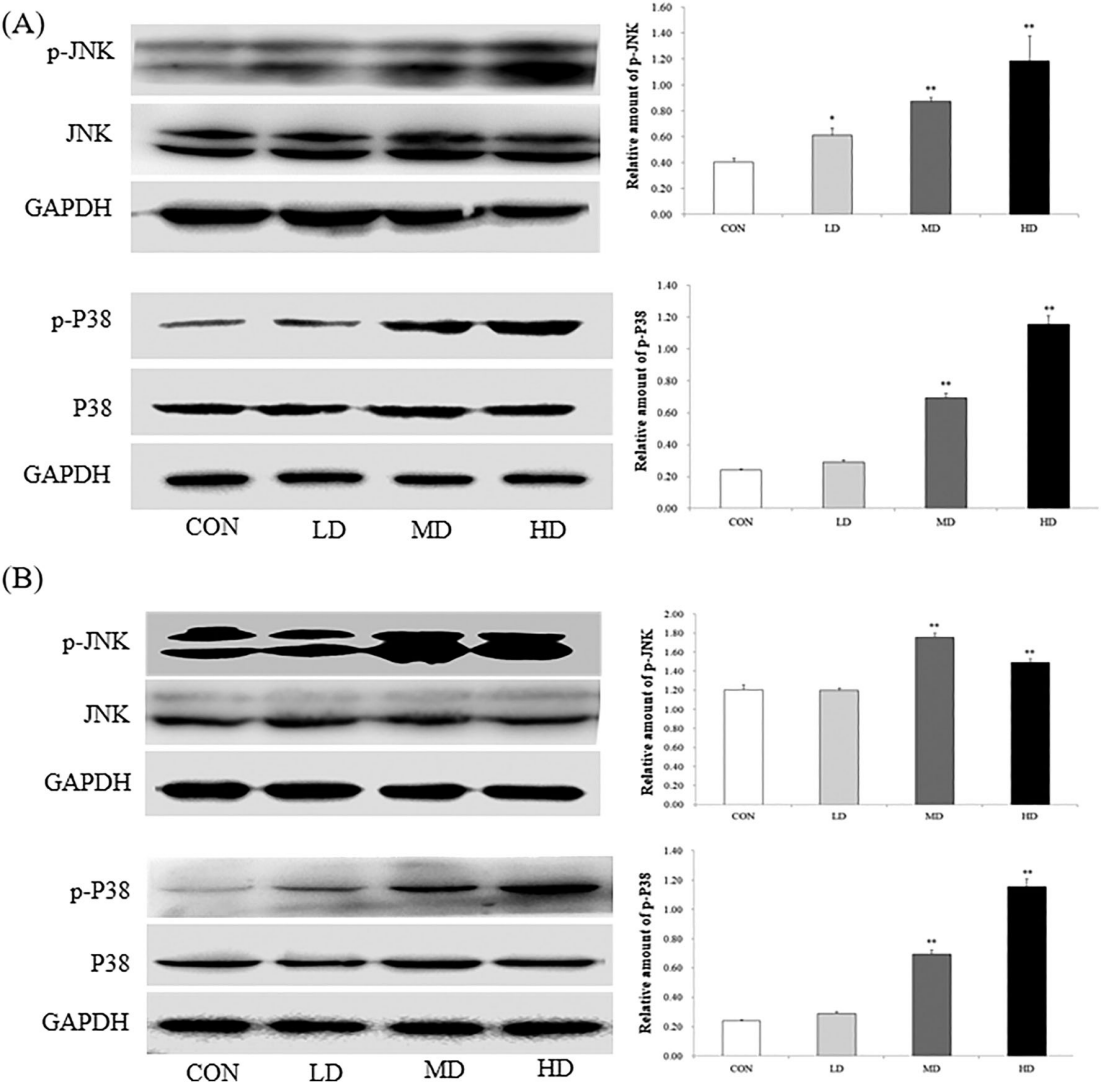
Effect of ATO exposure on mitogen‐activated protein kinase pathway. A, 12 h. B, 24 h. Activation of JNK and P38 in lung after ATO exposure 12 h and 24 h. Representative blots of the activation of JNK and P38 in lung. Density ratios of the activation of JNK and P38 were first calculated. Data represent mean ± SD (*n* = 3), *P < .05 compared with CON; **P < .01 compared with CON. ATO, arsenic trioxide; CON, control group; GAPDH, glyceraldehyde‐3‐phosphate dehydrogenase; HD, high‐dose group; JNK, c‐Jun N‐terminal kinase; LD, low‐dose group; MD, medium‐dose group

Figure 4A showed that, compared with the CON group, a significant increase was observed in the phosphorylated JNK (p‐JNK) in the LD group after exposure to ATO 12 hours (*P* < .05), and was more obvious in the MD and HD groups (*P* < .01). Meanwhile, the levels of phosphorylated P38 (p‐P38) were significantly increased in both the MD and HD groups (*P* < .01), and this effect was dependent on the exposure concentration. Figure 4B shows that, after exposure to ATO 24 hours, both the p‐JNK and p‐P38 were significantly increased in the MD and HD groups (*P* < .01).

## 4 DISCUSSION

The widespread coal combustion, nonferrous metal smelting and use of chemicals containing As, has led to high concentrations in the air and large amounts of As‐bearing particles can penetrate the lungs by breathing and subsequently enter the body. Keil and Richardson (2017) found that elimination of As exposure in workspaces would prevent 22 deaths by age 70 years per 1000 workers (95% confidence interval: 10, 35).

The change in the water content ratio of the lungs is caused by pulmonary edema or fluid exchange dysfunction between the blood vessels and tissues, causing fluid retention and gas exchange barriers with decreased lung compliance. The results showed that ATO exposure increased the lung water content ratio significantly, and the degree and duration of increase were exposure concentration‐dependent. Similarly, Tournel et al. (2011) found that acute ATO poisoning causes hemorrhagic pulmonary edema. Presumably, because As is in group 15 (Va) of the periodic table, during energy metabolism, As affects the formation of high‐energy phosphate bonds, thereby hindering cell energy metabolism. Moreover, As^3+^ combined with and inactivates a variety of important mercapto enzymes. This process inhibits the Na‐K pump function, leading to water sodium retention and an increased water content ratio (Shen, Li, Cullen, Weinfeld, & Le, 2013).

BALF can be used to obtain the alveolar protein concentration and inflammatory cell information that reflects the severity of lung inflammation, pulmonary cell membrane permeability changes and the degree of cell membrane damage. In this study, we found that the total protein concentration in the BALF after ATO exposure was significantly increased, and the effect was exposure concentration‐dependent. ATO exposure has been suggested to cause pulmonary inflammation, resulting in increased alveolar‐capillary permeability and cell lysis, and considerable protein and inflammatory cell exudation. At the same time, they were dose‐dependent (Umeda et al., 2013).

Pathological examinations can aid in the clinical diagnosis of disease, enhance the understanding of the nature of diseases and the trend of development, and facilitate the determination of the disease prognosis. H&E staining showed that after ATO intratracheal instillation, inflammatory damage occurred mainly in the alveolar structure. There was obvious pulmonary interstitial thickening, inflammatory cell infiltration, alveolar wall edema and alveolar wall thickening in a short time, which then gradually decreased. Moreover, the level of damage was significantly exposure concentration‐dependent. Guha Mazumder (2007) and von Ehrenstein et al. (2005) both found that ATO exposure in drinking water is also related to respiratory dysfunction as a potent respiratory toxicant. De, Majumdar, Sen, Guru and Kundu (2004) found that chronic ATO poisoning can lead to pulmonary obstructive lung injury, and the degree of injury is related to the degree of ATO poisoning. Based on the above results, it can be concluded that there is a correlation between ATO exposure and respiratory restriction, which may be caused by cell membrane edema.

Previous studies showed that after ATO exposure, the white blood cell, lymphocyte, neutrophil and monocyte counts decreased, but the ratio of lymphocytes increased. Ola‐Davies and Akinrinde (2016) also found that exposure to ATO decreases the leukocyte count. Furthermore, ATO likely inhibits hematopoiesis in the bone marrow by reducing the differentiation of blood cells. However, different degrees of inhibition of various differentiation pathways increases the ratio of lymphocytes. On the other hand, ATO exposure may cause aseptic inflammation and stimulate lymphocyte proliferation, leading to changes in leukocyte composition.

TNF‐α plays an important role in the regulation of inflammation and immunity and is an important proinflammatory cytokine and antitumor factor. IL‐6, an early proinflammatory factor, has an immunomodulatory effect and its increase is associated with acute infection (Baumann et al., 2016; Moshage et al., 1988). IL‐10 inhibits the release of inflammatory mediators from mononuclear macrophages and thereby inhibits the secretion of TNF‐α and IL‐6, and prevents inflammatory reaction progression. A significant increase in TNF‐α, IL‐6 and IL‐10 were observed in the study groups after ATO exposure. Bahari and Salmani (2017) also found that the gene expression of *TNF‐α* was significantly increased following exposure to the environmental dose of ATO. This phenomenon may occur because once ATO enters the lungs, TNF‐α as a proinflammatory cytokine mediates a cascade of immune responses. Our results suggest that the ATO‐induced increase of IL‐6 levels in rats led to inflammatory lung injury, and increased IL‐10, as an anti‐inflammatory factor, regulated the inflammatory response mediated by TNF‐α and IL‐6, to prevent the unlimited expansion of the immune defense system (Oberholzer, Oberholzer, & Moldawer, 2000). In addition, IL‐10 can also promote the healing process of injured tissues caused by infection or inflammation (Ouyang, Rutz, Crellin, Valdez, & Hymowitz, 2011). Therefore, our findings indicate the possibility of immune regulation and infection caused by ATO exposure. The findings have produced certain reference values that can assist in further study.

The mitogen‐activated protein kinase (MAPK) pathway is one of the most important pathways in the eukaryotic signal transduction network and plays a key role in gene expression regulation and cytoplasmic functional activities (Waskiewicz & Cooper, 1995). Among the signaling molecules of the MAPK pathway, JNK and P38 play an important role in the stress response, such as inflammation and cell apoptosis. JNK and P38 were found to be activated after exposure to ATO, and the degree of activation was dependent on the exposure concentration. Binet and Girard (2008) also found that ATO activates the JNK and P38. Lag et al. (2016) found that the activity of MAPK P38 appeared critically to mediate IL‐6 responses. These observations further proved that ATO exposure causes inflammatory responses in rats, and the MAPK pathway is one of the possible mediators of this effect.

In conclusion, intratracheal instillation of ATO in rats can cause the restriction to aeration, which was due to pulmonary edema and interstitial thickening, presumably due to the inflammatory injury induced by ATO exposure. Furthermore, the degree of injury was dependent on the dose of ATO. Our results indicate that acute atmospheric As exposure in humans, particularly the acute As exposure under extremely heavy haze, is associated with a risk of inflammatory lung injury, which is a health concern that deserves more attention.

## 5 LIMITATIONS AND FUTURE RESEARCH

The focus of our study is on the effects on the respiratory system of As exposure in the air, so we did not consider the factors of As particulate deposition in the lungs when determining the dose, which may cause the exposure dose to exceed the actual As content of the environment. However, this dose is still lower than the level of the As in the occupational population. In fact, understanding the effects of As on the respiratory system in the air has practical implications for explaining lung damage caused by air pollution. At the same time, the method of exposure we chose is one of the common exposure methods for respiratory exposure, i.e., tracheal instillation. Although tracheal instillation is complicated, it has the advantage of providing accurate exposure doses. In the future, we will study the mechanism of As in lung injury caused by air pollution and the impact on biological infections.

## CONFLICT OF INTEREST

The authors have no conflicts of interest to declare.

## AUTHOR CONTRIBUTIONS

Chao Liu and Qiang Wang designed the study. Mingxing Su, Haiying Wang, Congsong Sun and Chunyu Yuan performed the experiments. Mingxing Su discussed, drafted and wrote the paper.

## FUNDING INFORMATION

The authors acknowledge financial support from the National Key R&D Program of China (grant no. 2017YFF0211202), the Army Logistics Research Plan of China (grant no. AEP14C001) and the National Natural Science Foundation of China (grant no. 81472478).
